# Emergency burr-hole evacuation for subacute SDH with uncal herniation: a case report of near-complete neurological recovery despite bilateral occipital infarction

**DOI:** 10.1097/RC9.0000000000000411

**Published:** 2026-04-07

**Authors:** Shahin Naghizadeh, Maryam Zohrabi-Fard, Roozbeh Tavanaei, Saba Mirzaei, Parsa Avvalabadi, Saeed Oraee-Yazdani

**Affiliations:** a Student Research Committee, School of Medicine, Shahid Beheshti University of Medical Sciences, Tehran, Iran; b Functional Neurosurgery Research Center, Research Institute of Functional Neurosurgery, Shohada Tajrish Neurosurgical Center of Excellence, Shahid Beheshti University of Medical Sciences, Tehran, Iran

**Keywords:** case report, emergency burr-hole evacuation, fixed dilated pupils, occipital lobe infarction, subacute subdural hematoma, uncal herniation

## Abstract

**Introduction and importance:**

Subacute subdural hematoma (SDH) can remain clinically silent before progressing to fatal herniation. Bilateral fixed dilated pupils in the setting of SDH are traditionally regarded as a near-terminal sign, associated with poor outcomes. Reports of near-complete neurological recovery despite established uncal herniation remain exceedingly rare. This case highlights the importance of rapid decompression as a “brain code” intervention in potentially reversible mass lesions.

**Case presentation:**

A previously healthy 47-year-old woman presented with a 2-week history of worsening headaches and intermittent confusion. Initial CT imaging revealed a left frontoparietal subacute SDH with a significant midline shift. While preparing for emergent evacuation, she experienced an abrupt neurological collapse, developing a Glasgow Coma Scale of 3 and bilateral fixed dilated pupils. Immediate burr-hole evacuation resulted in rapid brain relaxation. Postoperatively, she regained full consciousness with a near-complete neurological recovery; however, an MRI confirmed bilateral occipital infarctions resulting in permanent cortical visual impairment.

**Clinical discussion:**

This case emphasizes that timely intervention can reverse the physiology of herniation before irreversible brainstem ischemia occurs. While bilateral fixed pupils have been regarded as a sign of futility, aggressive early decompression may provide meaningful recovery in carefully selected patients, especially when deterioration is witnessed and the time to surgery is minimal.

**Conclusion:**

Rapid burr-hole evacuation can reverse impending uncal herniation from subacute SDH and result in unexpected but meaningful neurological outcomes in selected patients when deterioration is witnessed and decompression is performed ultra-early. Fixed dilated pupils should not automatically preclude aggressive intervention when the underlying cause is promptly treatable.

## Introduction

Subdural hematoma is a neurosurgical emergency that requires prompt diagnosis and intervention to prevent irreversible neurological injury^[^1^]^. Acute and subacute subdural hematomas (SDH) can cause elevated intracranial pressure (ICP) and brain herniation if not promptly treated^[^1^]^. In particular, uncal herniation is among the most severe forms of herniation, often compressing the brainstem and rapidly leading to coma or death^[^2–4^]^. Clinically, patients may exhibit bilateral mydriasis due to third cranial nerve and brainstem compression, along with extensor posturing, Cushing’s triad, and GCS deterioration, indicating a critical “brain code” situation^[^2,3,5^]^.

HIGHLIGHTS

- Rapid burr-hole decompression reversed an impending uncal herniation.
- Near-complete neurological recovery occurred despite bilateral fixed pupils.
- A bilateral occipital infarction resulted in permanent cortical visual loss.
- Ultra-early decompression may challenge premature futility judgments in witnessed herniation.
- This case supports aggressive management of “brain code” herniation syndromes.

Historically, bilateral fixed and dilated pupils (BFDPs) in the context of traumatic brain injury or acute hemorrhage have been considered an almost certain harbinger of fatal outcome or devastating neurological disability^[^6^]^. In a meta-analysis of patients with traumatic hematomas and bilateral fixed pupils, only 6.6% of those with subdural hematomas had a good functional outcome, compared to over 50% in cases of epidural hematomas^[^6^]^. Consequently, many clinicians historically deemed bilateral fixed pupils as a sign of futility in SDH, often opting not to pursue surgical treatment^[^6^]^.

However, emerging evidence and case reports have challenged this nihilistic approach. Rapid surgical decompression can occasionally yield survival and even favorable recovery in carefully selected cases^[^7–9^]^. Recent retrospective analyses indicate that aggressive management of herniation syndromes, including immediate osmotherapy and decompressive surgery, can result in meaningful recovery in a subset of patients who would previously have been considered nonsalvageable^[^10^]^. These findings underscore a critical point: if intervention occurs before irreversible brainstem ischemia develops, the herniation physiology may still be reversible. Nonetheless, reports of full neurological recovery after bilateral fixed pupils and uncal herniation remain exceedingly rare in the literature. Here, we present a remarkable case of a patient who manifested deep coma and bilateral mydriasis from a subacute SDH with incipient uncal herniation, yet made a near-complete recovery following an emergency burr-hole decompression. We discuss the unique features of this case in the context of existing literature, highlighting the clinical implications for prognostication and management of “brain code” herniation scenarios. This case report has been reported in accordance with the updated Surgical CAse REport (SCARE) 2025 criteria^[^11^]^.

## Case presentation

A 47-year-old previously healthy woman presented with a 2-week history of progressively worsening headaches and several recent episodes of confusion. She denied any history of recent or remote head trauma, including minor or trivial injury. She was not taking anticoagulant or antiplatelet medications and reported no history of alcohol consumption or substance abuse. Initial laboratory investigations, including coagulation studies, were within normal limits, with a normal international normalized ratio, prothrombin time, activated partial thromboplastin time, and platelet count. On admission (26 May 2025), she was drowsy but oriented with a Glasgow Coma Scale (GCS) score of 13, and her neurological examination showed no focal motor or sensory deficits. The patient had no significant past medical, surgical, or medication history and was not on any regular treatment.

**Figure 1.**
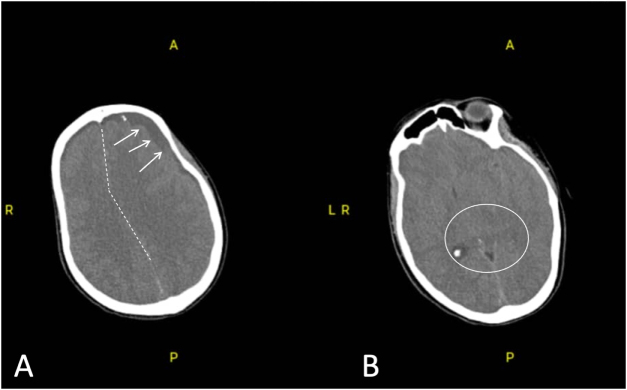
Preoperative non-contrast cranial CT. (A) A non-contrast axial CT scan demonstrates a large left frontoparietal subacute subdural hematoma with mixed-density (predominantly hypodense) components, measuring approximately 20 mm in maximal thickness (arrows). There is a significant mass effect with effacement of the left convexity sulci, compression of the left lateral ventricle, and a rightward midline shift of approximately 12 mm (dashed line). (B) A more posterior axial section again shows the left-sided subacute subdural hematoma with persistent mass effect and rightward midline shift. The basal cisterns appear compressed (encircled), consistent with early transtentorial herniation. All images were fully anonymized.

Non-contrast CT demonstrated a large left frontoparietal SDH measuring 20 mm in thickness, with a 12 mm midline shift and compression of the left lateral ventricle (Fig. 1). Preoperative optimization focused on rapid airway protection and hemodynamic stabilization. The patient was kept *nil per os* since arrival, intravenous access was secured, and preoperative laboratory tests were obtained. No adjustments to existing medications, lifestyle optimization, or psychological intervention were required due to the emergent nature of the deterioration.

While awaiting surgical preparation, the patient experienced an abrupt loss of consciousness and apnea approximately 50 min after arrival, with a drop in GCS to 3. BFDPs were documented shortly after deterioration, prior to transfer to the operating room. Endotracheal intubation was performed immediately, and the patient was transferred directly to the operating room. Surgical incision and decompressive burr-hole evacuation were initiated within approximately 5–10 min of pupillary fixation, resulting in an estimated total duration of bilateral fixed pupils of roughly 5–10 min. Pupillary fixation was therefore documented before incision and rapidly reversed following decompression.

Timeline: The patient first developed progressive headaches on 12 May 2025, followed by episodes of confusion in the days preceding admission, and presented to the emergency department on 26 May 2025. Acute neurological deterioration occurred approximately 50 min after arrival, prompting immediate surgical intervention without delay. Postoperative neuroimaging was performed on 27 May 2025, and the patient was discharged home on 01 June 2025. The clinical timeline is summarized in Table 1.

**Table 1:** Timeline of clinical events.

Date	Event
12 May 2025	Progressive headache begins
23–25 May 2025	Intermittent confusion episodes appear
26 May 2025	Patient presents to ED; CT reveals large subacute SDH
26 May 2025	Sudden neurological collapse; bilateral fixed dilated pupils
26 May 2025	Emergency burr-hole evacuation performed
27 May 2025	Postoperative CT + MRI; occipital infarction confirmed
01 June 2025	Discharged home neurologically intact with visual impairment
27 August 2025	Formal ophthalmologic evaluation confirms bilateral cortical visual field loss

Key clinical milestones from symptom onset to postoperative follow-up.

ED, emergency department; SDH, subdural hematoma; GCS, Glasgow Coma Scale; POD, postoperative day.

## Operative technique

At the time of neurological deterioration, airway protection and hemodynamic stabilization were immediately prioritized. Hyperosmolar therapy with intravenous mannitol was administered promptly following pupillary fixation. The procedure was performed under general anesthesia with the patient positioned supine and the head slightly elevated in a neutral position. Standard chlorhexidine-based skin preparation was applied. Two burr holes were created over the left frontal and parietal convexities. Upon dural opening, a tense dura was encountered, and approximately 120 mL of dark, liquefied subdural hematoma was released under pressure, producing immediate brain relaxation.

A closed subdural drain (standard silicone catheter) was inserted and left in place for 48 h postoperatively to allow continued decompression. The incisions were closed in a standard fashion without the need for craniectomy. Per institutional protocol, perioperative antibiotic prophylaxis and intravenous analgesia were administered. Given the immediate availability of surgical decompression and the extremely short interval between deterioration and incision, prolonged hyperventilation and vasopressor support were not required.

Rapid mechanical decompression was considered the most definitive and time-critical intervention for restoration of cerebral perfusion. The surgery was performed by a board-certified attending neurosurgeon at a tertiary referral neurosurgical center with routine experience in emergency cranial procedures and burr-hole decompression for subdural hematoma. No hybrid or multi-specialty collaboration was required for this intervention.

## Postoperative course and follow-up

**Figure 2.**
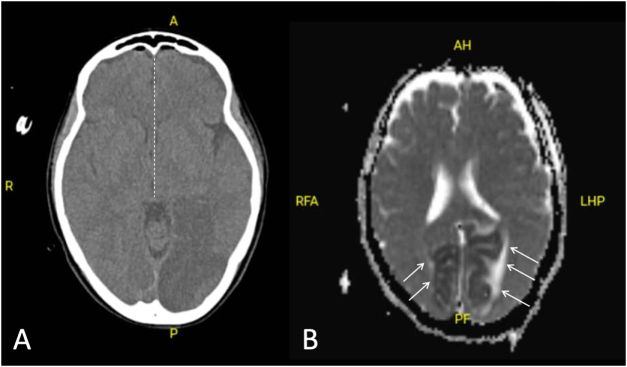
Postoperative imaging findings. (A) An axial non-contrast CT scan obtained several hours after burr-hole evacuation demonstrates re-expansion of the left cerebral hemisphere. The midline shift has resolved (dashed line), indicating reversal of transtentorial herniation. (B) Axial MRI diffusion-weighted imaging and corresponding apparent diffusion coefficient (ADC) map demonstrating symmetric diffusion restriction involving both occipital lobes (arrows), consistent with acute bilateral posterior cerebral artery territory infarction. All images were fully anonymized.

Postoperatively, the patient was managed in the intensive care unit with routine neurocritical care measures, including maintenance of adequate cerebral perfusion pressure with systolic blood pressure targets between 140 and 160 mmHg, normocapnia on arterial blood gas analysis, and sedation titrated to allow early neurological assessment. Within the first postoperative hour, the patient’s pupils became sluggishly reactive, with mild anisocoria that gradually improved over the following hours. Immediate postoperative CT imaging confirmed near-complete reversal of the midline shift and re-expansion of the basal cisterns, radiographic correlates strongly suggesting restoration of brainstem perfusion following decompression (Fig. 2A). Her level of consciousness steadily improved in the intensive care unit, and she was successfully extubated on postoperative day (POD) 1 with a GCS of 15. Although neurologically intact, she reported persistent blurring and constriction of her visual fields. Postoperative magnetic resonance imaging (MRI; ADC) performed on 27 May 2025 confirmed near-complete bilateral occipital lobe infarction (Fig. 2B). The closed subdural drain was removed on POD2, and the patient was mobilized under standard physiotherapy supervision. She was discharged home on 1 June 2025, fully oriented and independent in activities of daily living, with stable visual impairment – a discharge timeframe consistent with the expected length of stay after burr-hole evacuation.

Follow-up assessments were undertaken in person at the neurosurgery outpatient clinic of the same tertiary neurosurgical center, initially at 1 week, then at 1 month, and again at 3 months postoperatively, conducted by the operating neurosurgeon with collaboration from the ophthalmology service for visual rehabilitation and prognostication. Follow-up evaluation included clinical neurological examination and formal Humphrey 24-2 automated visual field testing.

Postoperative instructions included avoidance of heavy lifting and Valsalva-inducing activities for 6 weeks, wound care monitoring, and visual rehabilitation counseling. The patient demonstrated full adherence to these recommendations without intolerance or complications.

At the 3-month review, formal ophthalmologic assessment was performed, including automated visual field testing with Humphrey perimetry, which demonstrated global end-stage depression (left eye: visual field index (VFI) 1%, mean deviation (MD) −32.29 dB; right eye: VFI 3%, MD −31.36 dB), consistent with permanent cortical visual impairment resulting from bilateral posterior cerebral artery territory infarction. The preservation of normal optic discs and retinal nerve fiber layers supported a cortical rather than optic nerve pathology. This pattern aligns with the physiological convergence of monocular input in the visual cortex, explaining the symmetric bilateral deficit.

Despite this permanent visual loss, the patient’s overall neurological recovery was considered excellent given the preoperative signs of brainstem dysfunction, bilateral fixed dilated pupils, and impending uncal herniation. Favorable outcomes in similar contexts remain exceedingly rare, with less than 7% of patients with subdural hematoma and bilateral fixed pupils achieving meaningful recovery as reported in previous literature.

The bilateral occipital infarction was classified as a Clavien–Dindo Grade II postoperative complication, managed conservatively without reoperation and requiring scheduled ophthalmologic follow-up. No additional complications, readmissions, wound issues, or medical adverse events occurred within 30 days or during continued follow-up. Given the rarity of the complication and the atypical favorable neurological outcome, the case was formally reviewed within the departmental morbidity and mortality meeting.

## Discussion

SDH may remain clinically mild for days before reaching a critical threshold that precipitates abrupt neurological deterioration. In such cases, progressive intracranial volume compensation may abruptly fail, resulting in rapid ICP elevation and transtentorial herniation. This case illustrates how even a subacute hematoma can transition into a time-critical neurosurgical emergency, underscoring the importance of close monitoring and early recognition of deterioration in patients with sizable subdural collections.

BFDPs are generally regarded as an ominous clinical sign, typically reflecting midbrain compression and ischemia during transtentorial herniation^[^6,12^]^. Historically, outcomes in patients with subdural hematoma and bilateral pupillary non-reactivity have been extremely poor^[^8^]^ with reported rates of favorable functional recovery below 10%, in stark contrast to epidural hematomas, where outcomes are substantially better following timely evacuation^[^6^]^. As a result, pupillary fixation in this context has often been interpreted as a marker of futility.

However, this case challenges such deterministic prognostication by demonstrating that neurological recovery may still be possible when deterioration is witnessed, and decompression is performed at an ultra-early stage. The exceptionally short duration of pupillary fixation and the immediate reversal of mass effect suggest that brainstem perfusion was restored before irreversible ischemic injury occurred. Although causality cannot be definitively established from a single case, the temporal relationship between rapid decompression and neurological improvement supports the concept that pupillary fixation does not invariably signify permanent brainstem damage in carefully selected, time-critical scenarios. This aligns with recent reports that support time-to-surgery as an independent predictor of functional recovery in traumatic acute subdural hematoma, with thresholds of approximately 3 h in comatose patients^[^13^]^.

To our knowledge, reports of meaningful recovery following uncal herniation with bilateral fixed pupils remain exceedingly rare, particularly in the setting of subdural hematoma. Most previously described cases with favorable outcomes involve epidural hematomas or spontaneous improvement prior to surgical intervention. Compared with these reports, the present case is distinguished by the immediacy of surgical decompression during active herniation and the near-complete neurological recovery achieved despite profound preoperative neurological compromise. Jain *et al* reported a unique case of a spontaneous acute SDH with prolonged bilateral unreactive pupils that spontaneously partially regressed; the patient improved to GCS 15 after eventual surgery, marking the first such recovery in a non-traumatic SDH^[^8^]^. In that case, the herniation signs abated over several hours, possibly due to auto-decompression of the hematoma, before surgical evacuation.

In contrast, our patient had active intervention during the herniation and an almost immediate reversal of neurological deficit. The speed and completeness of her recovery appear exceptionally rare. Another report by Athanasiou *et al* described a trauma patient with 12 h of bilateral mydriasis who improved after decompressive craniectomy, but ultimately succumbed to medical complications^[^14^]^. Several landmark studies and guideline-based reviews have emphasized that timely surgical evacuation of acute subdural hematomas is critical to prevent secondary ischemia, brain herniation, and poor neurological outcomes. Early decompression, particularly within the first few hours after neurological deterioration, has repeatedly been shown to significantly improve survival compared with delayed intervention^[^15,16^]^. Our case further reinforces the importance of rapid decompression in preventing irreversible secondary injury.

It is also worth noting that our patient’s hematoma was in a subacute, partially liquefied phase and not accompanied by radiological or clinical evidence of severe primary parenchymal injury, which likely contributed to the favorable neurological outcome^[^17^]^. By contrast, traumatic acute SDH is frequently associated with extensive primary brain injury, a major determinant of outcome even when the hematoma is promptly evacuated^[^17,18^]^. In non-acute SDH, the underlying brain is typically compressed rather than structurally destroyed, so timely decompression can result in striking neurological improvement provided prolonged ischemia has not occurred^[^17^]^. While favorable outcomes are common in chronic SDH after burr-hole drainage, recovery in the context of active herniation – as in this case – is exceptionally uncommon^[^19–21^]^.

The bilateral occipital infarctions observed in this patient are most consistent with posterior cerebral artery territory ischemia occurring in the setting of transtentorial herniation. MRI, including diffusion-weighted imaging, demonstrated diffusion restriction involving both occipital lobes, a pattern classically associated with acute ischemic injury. No additional vascular imaging, such as computed tomography angiography or magnetic resonance angiography, was performed, which limits the ability to definitively exclude alternative vascular mechanisms. Nevertheless, the abrupt neurological deterioration, documented pupillary fixation, radiological evidence of mass effect with cisternal compression, and the characteristic bilateral occipital distribution strongly support transient posterior cerebral artery compromise secondary to herniation as the most plausible mechanism. Although a direct causal relationship cannot be conclusively established from a single case, the temporal association and imaging findings suggest herniation-related ischemia rather than embolic or intrinsic vascular disease.

Conceptually, this case aligns with the emerging paradigm of “brain code” management, in which acute herniation is treated as a neurological equivalent of cardiac arrest, demanding immediate intervention to restore perfusion before irreversible injury ensues. Just as delayed resuscitation in cardiac arrest results in poor outcomes, delays in decompression during herniation may convert potentially reversible injury into permanent neurological death.

## Strengths and limitations

### Strengths

This case highlights the potential reversibility of impending uncal herniation from an SDH when rapid decompression is performed, despite the presence of bilateral fixed dilated pupils, which are typically associated with irreversible brainstem injury and poor outcomes. The case provides high clinical impact learning, reinforcing “brain code” responsiveness and the importance of minimizing time-to-intervention. A second strength is the diagnostic clarity, supported by early postoperative CT and MRI correlating radiological reversal of mass effect with functional brainstem recovery. Additionally, the case carries cross-specialty relevance, offering insight for neurosurgeons, intensivists, neurologists, and emergency physicians, particularly regarding decision-making in scenarios traditionally considered futile. The case also demonstrates the value of early neuro-ophthalmologic evaluation in determining the anatomical origin of visual loss and counseling patients regarding recovery expectations.

### Weaknesses and limitations

The primary limitation of this case is that the favorable neurological recovery may not be universally generalizable, especially to patients with prolonged herniation, severe primary parenchymal injury, or delayed intervention. The emergent nature of the deterioration precluded standard preoperative investigations, structured visual field assessment, and multidisciplinary counseling before surgery. Although the complication of bilateral posterior cerebral artery–territory infarction was managed successfully in this patient, it represents a significant potential risk. It may result in profound disability in other individuals. Furthermore, because the patient experienced deterioration with immediate surgical access, the outcomes may not be replicable in resource-limited settings or in unwitnessed herniation events. These limitations highlight the need for carefully selected patient contexts and reinforce the need to balance aggressive intervention, while potentially life-saving, with realistic prognostic expectations and patient-centered decision-making.

## Conclusion

This case illustrates that near-complete neurological recovery may still be achievable in exceptional circumstances, even in the presence of bilateral fixed dilated pupils and impending uncal herniation due to SDH. When neurological deterioration is witnessed, and decompression is performed at an ultra-early stage, before the development of irreversible brainstem ischemia, restoration of intracranial physiology may permit meaningful recovery despite traditionally ominous prognostic indicators. Importantly, pupillary fixation in this context does not invariably imply permanent brainstem injury, particularly when the underlying pathology represents a rapidly reversible mass effect. At the same time, this case underscores that successful life-saving intervention does not preclude secondary ischemic complications, as posterior cerebral artery territory infarction and permanent cortical visual impairment may still occur despite timely decompression. Although such outcomes remain exceedingly rare and should not be overgeneralized, this experience supports an initially aggressive surgical rescue strategy in carefully selected, time-critical herniation scenarios, followed by reassessment once physiological stability has been restored.

## Patient’s perspective

I initially went to the hospital because of persistent headaches and increasing confusion, without realizing how severe my condition actually was. I do not remember the moment when I suddenly lost consciousness, but I was later told that my situation was life-threatening. I am deeply grateful for the rapid actions of the medical team, which saved my life. Although I have permanent visual limitations due to the occipital stroke, I am able to live independently and return to my daily activities. I consider my recovery a second chance at life, and I hope that my experience helps other patients receive timely care.

## Data Availability

The data that support the findings of this study are available from the corresponding author upon reasonable request.

## Artificial intelligence statement

Generative artificial intelligence (AI; ChatGPT by OpenAI, accessed online in November 2025) was used for language refinement, paraphrasing, and initial brainstorming of potentially relevant literature. All references suggested by AI tools were independently verified, critically appraised, and manually selected by the authors using primary source databases. AI was not used for data extraction, data analysis, result interpretation, or clinical decision-making. No patient-related clinical data, imaging, or identifiable information were provided to the system. All generated content was reviewed, fact-checked, corrected, and edited by the authors, who take full responsibility for the accuracy, relevance, and integrity of the final manuscript and reference list. The use of AI complied with institutional and ethical standards and did not involve any financial or commercial conflicts of interest with AI vendors.
